# Simulation-enhanced interprofessional education in surgery – a scoping review

**DOI:** 10.1186/s41077-026-00453-8

**Published:** 2026-07-23

**Authors:** Sean Stevens, Oscar Morice, Deborah Colville, Vijayaragavan Muralidharan, John Paige, Debra Nestel

**Affiliations:** 1 Department of Surgery, The University of Melbourne, Austin Precinct, Austin Health, 145 Studley Road, Heidelberg, VIC 3084 Australia; 2 Division of Surgery, Anaesthesia, and Procedural Medicine, Austin Health, 145 Studley Road, Heidelberg, VIC 3084 Australia; 3 Department of Surgery, School of Medicine, LSU Health New Orleans, 2021 Perdido Street, 8th floor, New Orleans, LA USA

**Keywords:** Simulation-enhanced interprofessional education (Sim-IPE), Simulation-based education (SBE), Simulation-based training (SBT), Interprofessional education (IPE), Health professions education (HPE), surgery

## Abstract

**Introduction:**

Simulation-enhanced interprofessional education (Sim-IPE) has been successfully used in various health professions and shown to improve communication and teamwork. Surgery, where preventable serious adverse outcomes are often attributed to issues with communication and teamwork, represents a compelling context for Sim-IPE. However, implementing Sim-IPE in surgery requires investment in faculty development, equipment and facilities while incurring the cost of reduced staff availability and theatre productivity during simulation activity. We conducted a scoping review to characterise how Sim-IPE in surgery is practised and researched.

**Methods:**

This scoping review followed the methodological framework described by Arksey and O’Malley. Reporting followed the PRISMA-ScR checklist. Electronic databases (Medline, EMBASE, Web of Science, PsycINFO and ERIC) were searched for records pertaining to Sim-IPE in surgery published after 1st January 2000 and in English.

**Results:**

The final analysis included 81 articles. Simulation scenarios showed a rich variety of combinations of professions, disciplines, training levels and settings. While most scenarios used manikins (*N* = 54, 75%), other modalities included simulated participants, cadavers, task trainers, computers, laparoscopic simulators, role play and animal models with many scenarios using multiple modalities (*N* = 20, 27.8%). Of studies reporting on evaluation outcome, almost half (*N* = 32, 47.1%) were limited to participants’ views of their learning experience and satisfaction with the program. Few studies reported using educational theory to inform Sim-IPE design and even fewer used theoretical frameworks in the research design. Facilitators for implementing Sim-IPE in surgery included a skilled faculty, institutional support and evidence of benefit. Barriers included lack of protected training time, restricted access to facilities and unreliable ongoing funding.

**Discussion:**

Studies described a rich variety of Sim-IPE scenarios, including multiple professions, disciplines, training levels, simulation modalities and settings, facilitating training in a broad range of skills reflective of the complexity of modern surgical care. While barriers such as time, funding, and cultural resistance to simulation are commonly reported in the simulation-based education literature, these issues may be amplified in surgery due to surgical service pressures and traditional hierarchies (e.g. surgeon as “captain of the ship”). Overcoming these barriers will likely require structural (policy, funding, scheduling) and cultural (leadership, team identity) change.

## Introduction

Simulation-based education (SBE) is widely practised within health professions education (HPE) [1]. A fundamental benefit of SBE is shifting clinicians’ focus from service provision to training, creating an environment conducive to education. Simultaneously, SBE facilitates experiential learning without patient risk [2]. Given the potential for high complexity, patient risk and practitioner stress, surgery appears particularly well suited to SBE approaches [3]. Predominantly, SBE has been used in surgery to improve medical knowledge and technical skills [3]. More recently, the use of SBE in surgery has expanded to train other core competencies of surgical practice such as communication, decision-making, teamwork and leadership (e.g. the Non-technical Skills for Surgeons (NOTSS) training system) [4–6]. While the term “non-technical skills” is widespread in the literature, the negative framing may be perceived to imply inferiority to technical skills [7]. As this is not the intent [8], this review adopts the term “professional skills” instead [9].

Interprofessional education (IPE) occurs when two or more health professions learn with, from, and about one another to improve collaborative, patient-centred practice and quality of care [10–13]. IPE has been shown to improve interprofessional collaboration and teamwork [14], reduced negative professional stereotypes [15] and enhanced patient care [16]. In recent decades, advocacy by prominent bodies such as the United States’ National Academy of Medicine and the World Health Organisation has helped drive the integration of IPE into HPE curricula [17–19]. Concurrently, the surgical community has emphasised the importance of professional skills as essential competencies in optimising patient-centred care [20–22]. Consequently, IPE has been increasingly implemented in surgical education and training programs and with favourable results [23–25].

Simulation has emerged as an effective method for conducting IPE (Simulation-enhanced Interprofessional Education or Sim-IPE) [12, 26, 27]. Sim-IPE has successfully been used in various health professions including nursing, medicine, physical therapy and pharmacy to improve professional skills [26–29]. Critique of Sim-IPE practice includes a need to incorporate IPE theoretical concepts into simulation design and strengthen evaluation methods [30].

Surgery, where serious adverse outcomes are often linked to issues with teamwork and communication [31–33], represents a compelling context for Sim-IPE. Evidence shows training in professional skills is associated with improved clinical outcomes in surgery [34]. However, implementing Sim-IPE in surgery may be onerous, requiring investment in faculty development, equipment and facilities while incurring the cost of reduced staff availability and theatre productivity during simulation activities [35]. This scoping review aims to map how Sim-IPE is currently practised, studied and reported within surgery and to identify gaps and opportunities for future practice and research.

## Methods

A protocol for this scoping review has been published [36]. We conducted this scoping review in accordance with the methodological framework described by Arksey and O’Malley (2005) and progressed by Levac (2010) [37, 38]. Reporting followed the Preferred Reporting Items for Systematic Reviews and Meta-Analyses Extension for Scoping Reviews (PRISMA-ScR) checklist and was further informed by the Joanna Briggs Institute (JBI) Manual for Evidence Synthesis and other best practice guidelines [39–41]. Protocol deviations are identified and justified below.

### Eligibility criteria

#### Population

We included records where the study population comprised healthcare professionals – either students, trainees or fully qualified. Eligible records must have included surgeons, surgical trainees or medical students learning surgical topics.

#### Concept

We included records that described both SBE and IPE as applied to the field of surgery. We defined SBE as a learning technique whereby a real-life task is performed in a modified learning environment [42]. We broadened the definition of ‘IPE’ to include individuals of the same profession (e.g. medical practitioners) who belonged to different specialties (e.g. surgery and anaesthesia). Surgery was defined as the medical specialty primarily responsible for the management of surgical conditions. Records pertaining to obstetrics and gynaecology were excluded unless another surgical specialty was also involved. This was to constrain the scope of this review to be conceptually coherent given obstetrics and gynaecology represents a large and distinct area of surgical and simulation-based educational practice with its own extensive interprofessional literature. We also excluded records where surgery was not central to the learning objectives, team composition or simulated task, including scenarios where surgical involvement was peripheral or incidental to a predominantly non-surgical scenario.

#### Context

We included records on Sim-IPE in surgery published after January 1, 2000, as preliminary searches did not identify suitable records prior to this time. Eligibility was limited to hospital settings (e.g. emergency department, operating theatre or surgical ward) where interprofessional team-based care involving surgery is most concentrated.

#### Evidence type

We included non-interventional articles (e.g. editorials, commentaries, perspective articles, review articles and book chapters) when they contributed substantively to understanding Sim-IPE in surgery beyond simple background description, for example through discussion of program design, implementation, evaluation or unique insights into the topic. We intended to include non-English language records but this proved impractical and such studies were excluded along with conference abstracts.

### Information sources

We searched five electronic databases for records pertaining to Sim-IPE in surgery: Medline, EMBASE, Web of Science, PsycINFO and ERIC.

### Search strategy

Our search strategy has been published (Table 1) [36]. We planned additional simulation journal and grey literature searches but the database search yielded so many records that we deemed further searches impractical and with likely marginal benefit.

**Table 1:** Search strategy for scoping review

Term (title/abstract)		Term (title/abstract)		Term (title/abstract)		Term (title/abstract)
Simulat*	AND	Educat* OR	AND	Inter-profession* OR	AND	Surg*
		Train* OR		Interprofession* OR		
		Teach OR		Inter-disciplin* OR		
		Learn OR		Interdisciplin* OR		
		Collaborat* OR		Multi-disciplin* OR		
		Cooperat* OR		Multidisciplin* OR		
		Assess*		Transdisciplin* OR		
				Multi-profession* OR		
				Multiprofession*		

The asterisk denotes wildcard truncation used in database searching, capturing all word endings after the preceding stem. Reproduced from Stevens 2024 [36], originally published in the Journal of Healthcare Simulation under a Creative Commons Attribution-Share Alike 4.0 International License

### Selection process

We imported all records into Covidence systematic review software (Veritas Health Innovation, Melbourne, Australia, www.covidence.org) for reference management and screening. Following exclusion of duplicates, two researchers (SS and OM) independently screened all records by title and abstract for eligibility, then reviewed full texts of included records. Differences were reconciled by consensus with input from a senior researcher (DN).

### Data charting

OM, DN, DC and SS charted the data using Covidence with SS reviewing all records. In addition to charting scenario and participant characteristics, we collected selected research-process and reporting variables including ethics approval, funding and conflict-of-interest reporting to help characterise how Sim-IPE in surgery is being studied and reported as an emerging field of scholarship. The data items charted are shown in Appendix 1.

### Data analysis and presentation

To synthesise and present our findings we analysed quantitative data (e.g. record and scenario characteristics) with simple descriptive numerical summaries and qualitative data using thematic analysis in an iterative process.

### Knowledge user consultation and statement of positionality

We followed the ACTIVE framework for “stakeholder” involvement [43] but replaced the word “stakeholder” with “knowledge user” given the sensitivity of the term. Knowledge users were recruited by invitation and often held multiple roles including general surgeons (SS, VM, JP), an ophthalmic surgeon (DC), a surgical trainee (OM), simulation practitioners (DN, SS, JP) and surgical education researchers (SS, DC, JP, DN). They contributed to shaping the review’s scope and approach, interpreting the findings and reviewing drafts of the manuscript. The initial clinical knowledge user group did not include a non-surgeon clinician, so interprofessional clinical input was later broadened through review of the draft manuscript by additional knowledge users including a nurse simulation educator and emergency department physician.

## Results

### Selection and characteristics

**Fig. 1:**
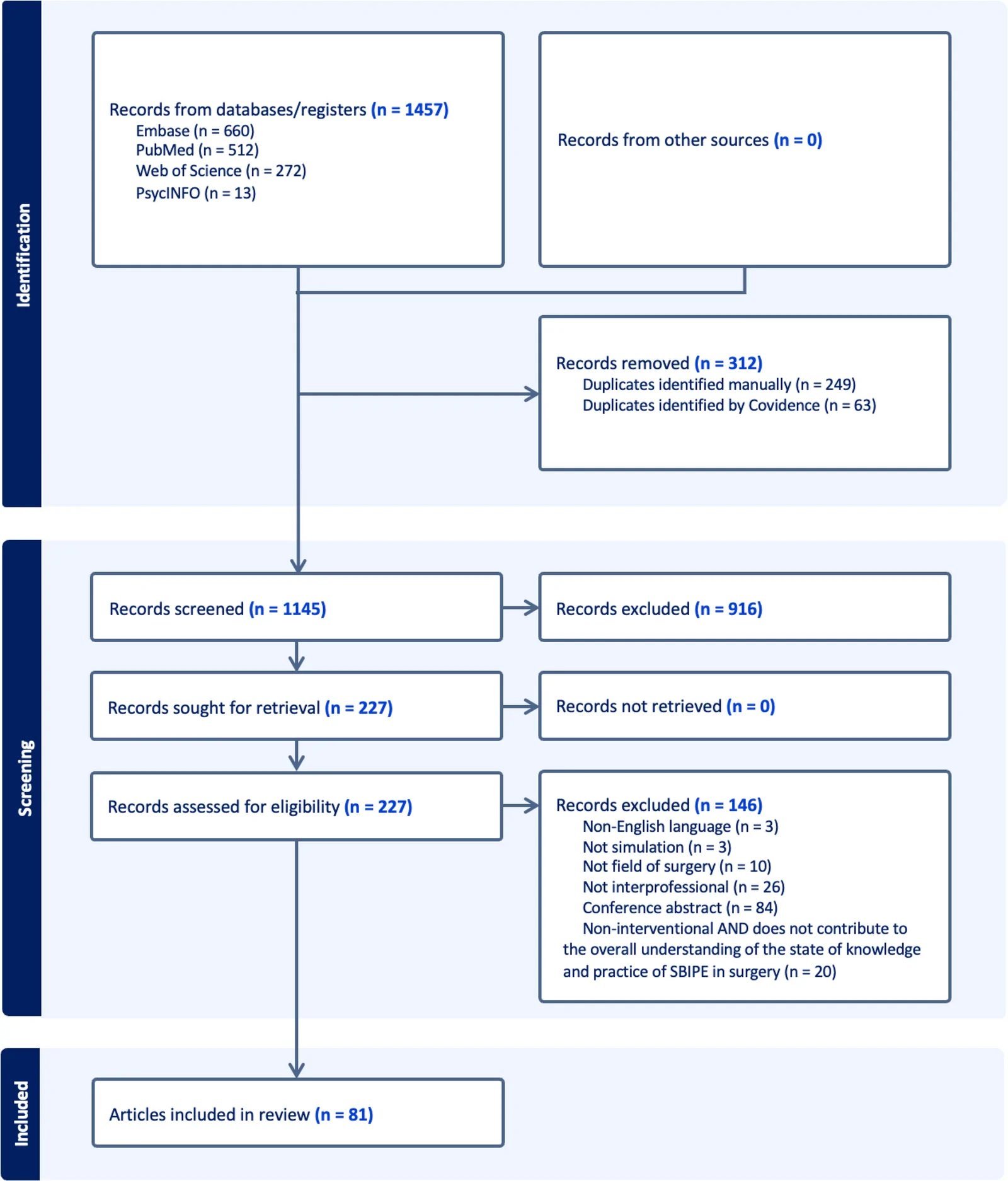
PRISMA flow diagram

**Fig. 2:**
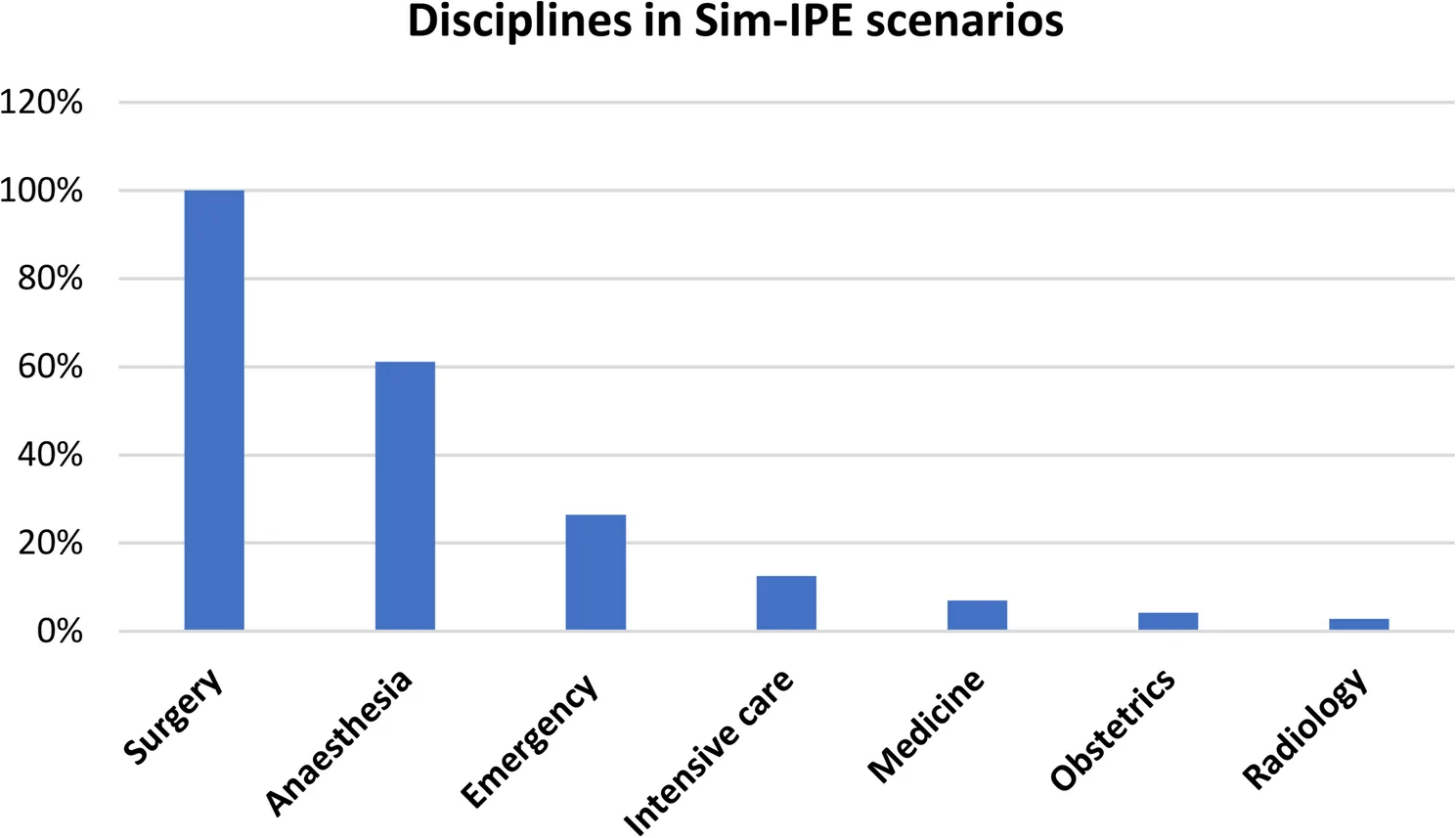
Proportional representation of disciplines in Sim-IPE in surgery scenarios

The search identified 1145 unique records (Fig. 1) of which 81 articles were included for analysis (Appendix 2). The characteristics of the selected articles are presented in Table 2. Annual publication numbers increased from 2000 to 2023 (Fig. 2). The earliest article was from 2007 and more than half (*N* = 42, 51.9%) were published between 2019 and 2023. The 81 articles originated from 11 countries; 90% of articles were from the Anglosphere with 58% from the USA. Ethical review was not applicable for 14 articles (i.e. reviews, book chapters, commentaries and simulation protocols). Among the 67 interventional studies, 49 (72.3%) reported institutional review board approval or exemption, while 18 (26.8%) did not report ethics review. Only 36 articles (44.4%) reported funding. Of those, 30 (83.3%) received academic funding only, 1 (2.8%) industry funding only and 5 (13.9%) both. Sixteen articles (19.8%) received no funding and 29 (35.8%) did not report funding status. Conflicts of interest were declared in 16 articles (19.8%, typically authors affiliations with simulation companies). No conflicts were declared in 37 articles (45.7%) and 28 (34.6%) did not report on conflict of interest. Regarding study design, 43 articles (53.1%) were non-randomised experimental studies. Sixteen (19.8%) were simulation scenarios without evaluation. Less common study designs included reviews (*N* = 7, 8.6%), randomised controlled trials (*N* = 4, 4.9%), qualitative (*N* = 4, 4.9%) and mixed methods studies (*N* = 3, 3.7%).

**Table 2:** The 81 articles included for analysis. Full references in Appendix 2

Author	Year	Country	Study design
Undre	2007	UK	Simulation scenario
Kozmenko	2008	USA	Non-randomised experimental study
Paige	2008	USA	Review
Paige	2009	USA	Non-randomised experimental study
Kinoshita	2010	Japan	Simulation scenario
Klipfel	2011	USA	Non-randomised experimental study
Steinemann	2011	USA	Simulation scenario
Ziewacz	2011	USA	Randomised controlled trial
Stevens	2012	USA	Simulation scenario
Boet	2013	Canada	Randomised controlled trial
Cumin	2013	New Zealand	Review
Dedy	2013	Canada	Survey
Seymour	2013	USA	Review
Ziesmann	2013	Canada	Simulation scenario
Arora	2014	UK	Non-randomised experimental study
Arriaga	2014	USA	Non-randomised experimental study
Doumouras	2014	Canada	Randomised controlled trial
Huser	2014	Germany	Non-randomised experimental study
Paige	2014	USA	Non-randomised experimental study
Shapiro	2014	USA	Non-randomised experimental study
van der Nelson	2014	UK	Non-randomised experimental study
Crozier	2015	Canada	Non-randomised experimental study
Hamilton	2015	USA	Non-randomised experimental study
Nicksa	2015	USA	Simulation scenario
Paige	2015	USA	Review
Phitayakorn	2015	USA	Non-randomised experimental study
Stefanidis	2015	USA	Simulation scenario
Weller	2015	New Zealand	Mixed methods: non-randomised experimental and qualitative study
Boet	2016	Canada	Randomised controlled trial
Gillman	2016	Canada	Simulation scenario
Sadideen	2016	UK	Qualitative research
Weller	2016	New Zealand	Non-randomised experimental study
Weller	2016	New Zealand	Qualitative research
Davis	2017	USA	Simulation scenario
Paige	2017	USA	Non-randomised experimental study
Joshi	2018	USA	Non-randomised experimental study
Lind	2018	USA	Non-randomised experimental study
Sullivan	2018	USA	Non-randomised experimental study
Wongsirimeteekul	2018	USA	Simulation scenario
Jowsey	2019	New Zealand	Qualitative research
Leithead III	2019	USA	Non-randomised experimental study
Long	2019	USA	Non-randomised experimental study
Paige	2019	USA	Book chapter
Paige	2019	USA	Simulation scenario
Rochlen	2019	USA	Non-randomised experimental study
Weis	2019	USA	Non-randomised experimental study
Chheang	2020	Germany	Non-randomised experimental study
Esparaz	2020	USA	Non-randomised experimental study
Hoang	2020	USA	Non-randomised experimental study
Long	2020	New Zealand	Qualitative
Lynch	2020	Australia	Review
Mai	2020	USA	Simulation scenario
Nataraja	2020	Myanmar	Simulation scenario
Weller	2020	New Zealand	Mixed methods: non-randomised experimental and qualitative study
Baste	2021	France	Non-randomised experimental study
Beaven	2021	UK	Non-randomised experimental study
Caldwell	2021	USA	Non-randomised experimental study
Fiorentino	2021	USA	Non-randomised experimental study
Gros	2021	USA	Non-randomised experimental study
Huffman	2021	USA	Non-randomised experimental study
Johnson	2021	Canada	Commentary
Kawaguchi	2021	USA	Review
Lucke-Wold	2021	USA	Simulation scenario
Lusty	2021	Canada	Simulation scenario
Towning	2021	UK	Non-randomised experimental study
Truong	2021	USA	Non-randomised experimental study
Dinh	2022	Vietnam	Non-randomised experimental study
Harrington	2022	USA	Non-randomised experimental study
Hartman	2022	USA	Non-randomised experimental study
Hopmann	2022	USA	Non-randomised experimental study
Jogerst	2022	USA	Non-randomised experimental study
Merritt-Genore	2022	USA	Non-randomised experimental study
Picard	2022	France	Mixed methods: non-randomised experimental and qualitative study
Sinyard	2022	USA	Review
Xue	2022	USA	Report
Escher	2023	Sweden	Non-randomised experimental study
Fitzgerald	2023	Australia	Non-randomised experimental study
Gallagher	2023	UK	Non-randomised experimental study
Horne	2023	USA	Non-randomised experimental study
Patki	2023	UK	Non-randomised experimental study
Zucco	2023	USA	Simulation scenario

### Sim-IPE in surgery scenario characteristics

The characteristics of the 72 articles that described *specific* simulation scenarios were analysed. Nine articles were excluded because of the general nature of their discussion of Sim-IPE in surgery. All simulation scenarios included doctors and the vast majority (*N* = 62, 86.1%) involved nurses. Just over half (38 scenarios, 52.8%) involved the combination of doctors, nurses and other workers. “Other workers” included a wide variety of professions and roles including physiotherapists, social work, pharmacists, paramedics, radiographers, theatre technicians, perfusionists, physician assistants, health care managers and administrative staff, blood bank, pastoral care and patient transport. The next most common combination was doctors and nurses (*N* = 24, 33.3%). A small proportion of articles involved doctors only (*N* = 9, 12.5%).

General surgery was the most commonly represented surgical specialty (*N* = 46, 63.9%), followed by cardiothoracic (*N* = 6, 8.3%), paediatric (*N* = 3, 4.2%), otolaryngology (*N* = 3, 4.2%), vascular (*N* = 2, 2.8%), plastic (*N* = 2, 2.8%) and neurosurgery (*N* = 1, 1.4%), with surgical specialty not being specified in 11 (15.3%) studies. After surgery, the most represented disciplines were from critical care; anaesthesia (*N* = 44, 61.1%), emergency medicine (*N* = 19, 26.4%) and intensive care (*N* = 9, 12.5%) (Fig. 2). Amongst the critical care disciplines, representation was by doctors, nurses or both doctors and nurses. Medicine (*N* = 5, 6.9%), obstetrics (*N* = 3, 4.2%) and radiology (*N* = 2, 2.8%) were the other disciplines represented and all by doctors.

Participants were from all training levels: students (*N* = 16, 22.2%), trainees (*N* = 50, 69.4%) and fully qualified professionals (*N* = 53, 73.6%). All possible combinations of these levels were seen; the most common was a mix of students, trainees and fully qualified professionals (*N* = 27, 37.5%), followed by fully qualified only (*N* = 16, 22.2%) (Table 3).

**Table 3:** Mix of participants in Sim-IPE in surgery scenarios by training level

Training level	*N* (%)
Student only	3 (4.2)
Trainee only	13 (18.8)
Fully qualified only	16 (22.2)
Student & trainee	3 (4.2)
Student & fully qualified	2 (2.8)
Trainee & fully qualified	8 (11.1)
Student & trainee & fully qualified	27 (37.5)
**Total**	**72 (100)**

**Fig. 3:**
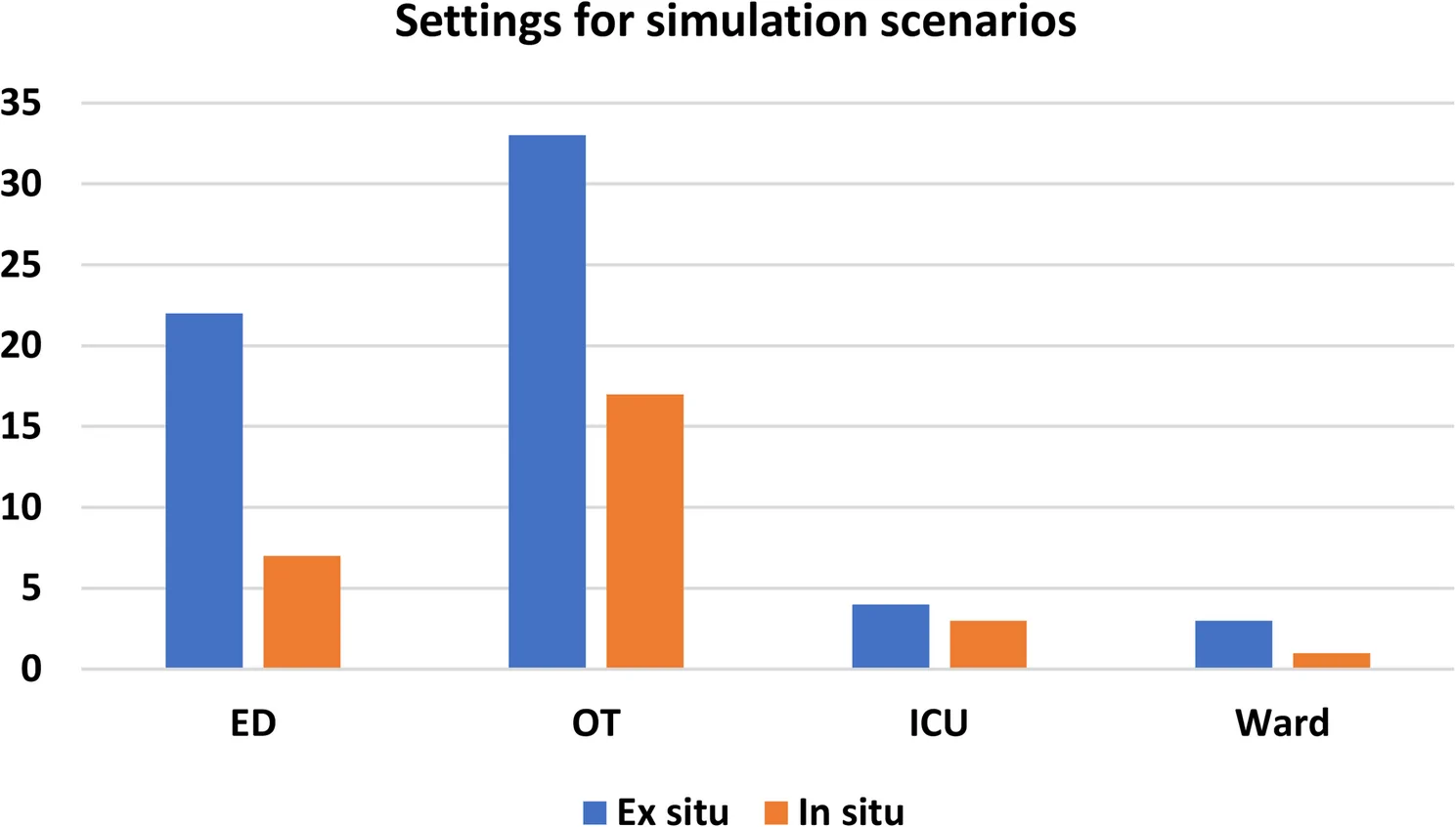
Clinical settings for simulation scenarios including ex situ (i.e. a simulated clinical space) or in situ (i.e. a real clinical space). ED = Emergency Department, OT = Operating Theatre, ICU = Intensive Care Unit (including high dependency unit and coronary care unit)

Most studies reported simulation scenarios that were ex situ (i.e. occurring in a simulated clinical space such as within a simulation centre) (*N* = 48, 66.7%), with 21 (29.2%) being in situ (i.e. occurring within a real clinical space) and three (4.2%) including both (Fig. 3). Scenarios were set in various environments, most commonly the emergency department (*N* = 27, 37.5%) and operating theatre (*N* = 48, 66.7%), with fewer in the intensive care unit (*N* = 6, 8.3%) and surgical ward (*N* = 4, 5.6%). Eleven studies (15.2%) used multiple settings.

Manikins were the most commonly used simulation modality (54 scenarios, 75%). Other modalities included simulated participants (*N* = 11, 15.3%), cadavers (*N* = 7, 10%), task trainers (*N* = 6, 8.3%), computers (*N* = 5, 6.9%), laparoscopic simulators (*N* = 4, 5.6%), role play (*N* = 3, 4.2%) and animal models (*N* = 2, 2.8%). Twenty simulation scenarios (27.8%) used multiple modalities.

Most scenarios were conducted at a single institution (*N* = 53, 73.6%). Eight (11.6%) involved two sites and eight (11.6%) involved three or more sites. Three articles did not report the number of sites involved.

Participant numbers per study ranged from 5 to 329 (median 48; interquartile range 76). Ten articles did not report the number of participants involved.

### Analysis, reported effects, educational theory and theoretical frameworks

No articles focused solely on individual (micro-level) outcomes. Most (*N* = 69, 92.07%) examined team (meso-level) outcomes while six (8.0%) studied hospital (macro-level) outcomes.

**Fig. 4:**
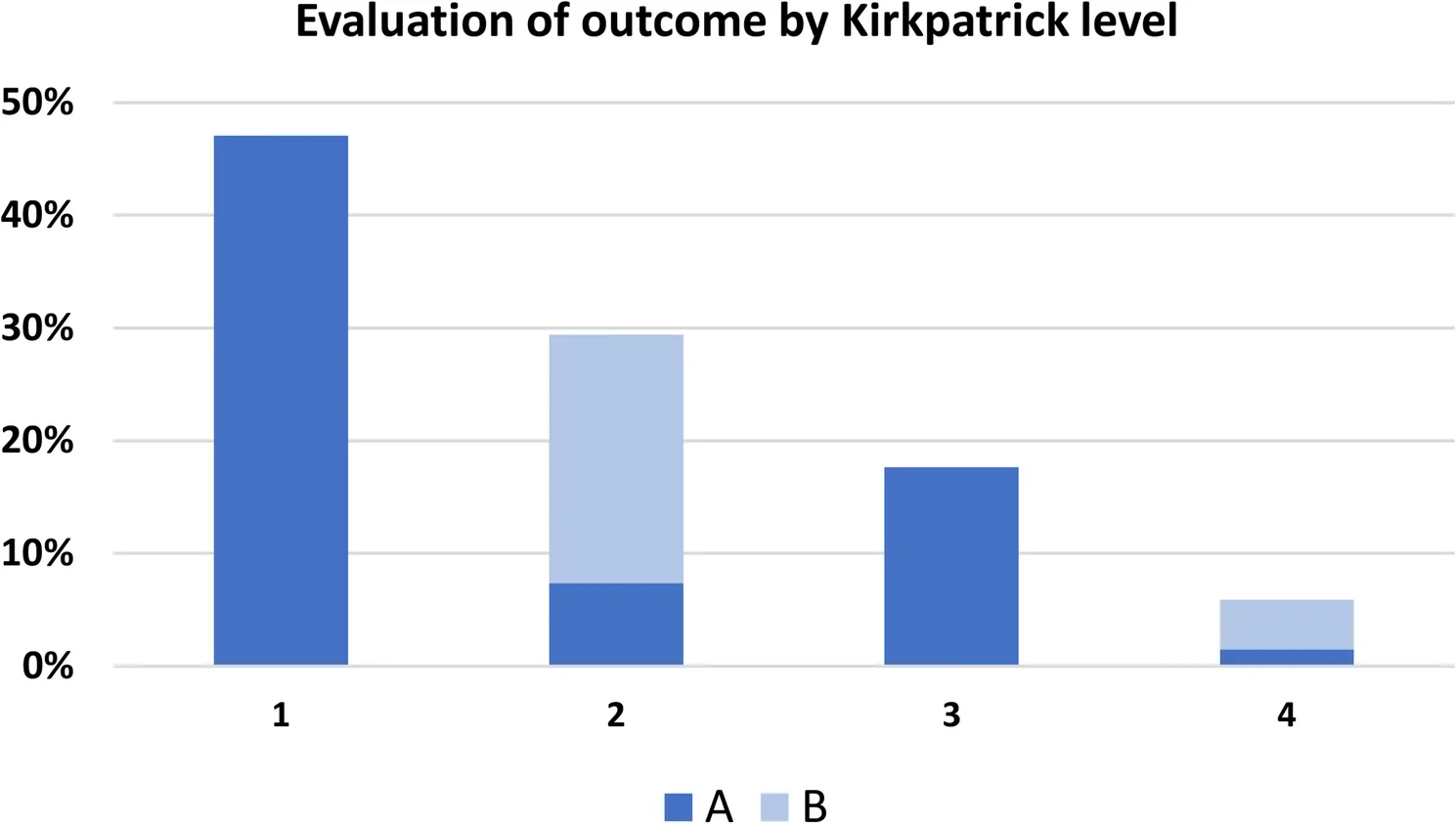
Modified Kirkpatrick level of evaluation outcome for interventional studies by percentage. Kirkpatrick level 1 *N* = 32, level 2 A *N* = 5, level 2B *N* = 14, level 3 *N* = 12, level 4 A *N* = 1, level 4B *N* = 4

Studies reporting evaluation outcomes (*N* = 68) were categorised by the Modified Kirkpatrick Model [44] (see Appendix 3) as shown in Fig. 4. Almost half the studies (*N* = 32, 47.1%) were limited to participants’ views of their learning experience and satisfaction with the program (Level 1). Other levels were less common; modification of participants’ attitudes (*N* = 5, 7.4%, Level 2 A); participants’ acquisition of knowledge or skills (*N* = 14, 20.6%, Level 2B); participants’ behaviour in the workplace (*N* = 12, 17.6%, Level 3); changes in organisational practice (*N* = 1, 1.5%, Level 4 A); and, benefits to patients (*N* = 4, 5.9%, Level 4B).

Most interventional studies reported positive effects from Sim-IPE. These were commonly described as improvement in knowledge, skills, team performance or communication behaviours such as “speaking up” and “expressing disagreement” [45, 46]. Occasionally, studies reported “negative” findings such as reduced confidence in leading trauma resuscitations after Sim-IPE [47]. Other notable findings included learning benefits for observers [48], use of Sim-IPE to shape professional attitudes among students [49], incremental gains across repeat scenarios [50] and application of Sim-IPE to just-in-time implementation of new clinical processes [51].

Educational theory was seldom discussed: 69 (85.2%) articles did not mention educational theory. Of the 12 (14.8%) that did, the most discussed was Ericsson’s theory of deliberate practice (*N* = 6, 7.4%), Kolb’s theory of experiential learning (*N* = 5, 6.2%) and Salas’ theory of effective teamwork (*N* = 2, 2.5%). Vygotsky’s theory of social constructivism and complex adaptive systems theory were each mentioned in a single article (1.2%). Three (3.7%) articles discussed more than one educational theory.

Similarly, the vast majority (92.6%) of articles did not mention a recognised theoretical framework. Of the six (7.4%) that did [52–57], only one (1.2%) article [52] explicitly used a theoretical framework (Rogers’ Diffusion of Innovation theory) to anchor their research’s design and analysis.

### Facilitators, barriers and major results

Numerous facilitators for implementing Sim-IPE in surgery were identified. Faculty with experience and skill in SBE were difficult to develop but highly valuable in designing and delivering Sim-IPE. Studies suggested that representation from participating professions and disciplines among faculty supported scenario design, participant buy-in and debriefing quality [58, 59]. Institutional support was crucial for facilitating funding, accessing clinical spaces, quarantining training time, and driving program organisation [57, 60]. Access to well-resourced facilities, appropriate equipment and clinical spaces was commonly reported as supporting scenario authenticity and delivery [4, 61, 62]. In addition, scenarios perceived as relevant to all participating groups appeared to enhance acceptability and learner buy-in [45, 60]. Finally, robust evidence of benefit, endorsement by national or international bodies and clear guidelines for developing Sim-IPE activities were seen as imperative for successful implementation [45].

In contrast, the most frequently cited barrier to implementing Sim-IPE in surgery was lack of quarantined time for training within regular working hours, with surgeons and surgical trainees particularly affected. However, there was concern that catering specifically to one profession or discipline was contrary to the ethos of team-based training. Another commonly reported barrier was developing faculty with the capability to both design and deliver Sim-IPE programs. Numerous studies reported the termination of successful Sim-IPE programs due to lack of funding once initial research grants ended. Cultural factors, such as prevailing attitudes toward both SBE and IPE, influenced the success of such programs, with some institutions reporting persistent scepticism towards IPE. Lastly, although recognised as beneficial, in situ simulation was particularly challenging to implement due to impacts on clinical service provision and revenue generation and this was especially so when conducted in the operating theatre.

## Discussion

Using a robust scoping review methodology, we mapped a rapidly expanding but uneven literature on Sim-IPE in surgery. The field is characterised by a rich diversity of scenarios, learner mixes, modalities and settings but is limited by inconsistent reporting, weak theoretical alignment and a focus on short-term, learner-level outcomes rather than organisational or patient outcomes.

A recurrent issue was variability in reporting of ethical approvals, funding sources and conflicts of interest. Given the emerging nature of Sim-IPE in surgery research, variability in standards of reporting is unsurprising and parallels early trends in SBE research that prompted the publication of reporting guidelines [63]. Notably, while these guidelines specifically recommend disclosure of funding sources and conflicts of interest, they omit guidance on ethical approval.

Research within HPE has been criticised for variability in ethical conduct and reporting [64]. Yet, numerous ethical concerns are known to exist within HPE research (e.g. participant/researcher power imbalances, preserving confidentiality and ensuring psychological safety) [64–66]. These concerns have led to dedicated guidelines on ethical practice in HPE research [67]. Despite these resources, uncertainty seemingly persists regarding when ethical review is required and in the differentiation between quality improvement (QI) and research. Interventional studies submitted to peer-reviewed journals for publication clearly fall under “research” rather than “QI” [68, 69]. Consistently applying established reporting checklists, clearly documenting ethical approvals and distinguishing QI from research will likely strengthen Sim-IPE in surgery’s credibility as the field matures.

Cook et al. (2008) categorised medical education research as descriptive (“what was done?”), justification (“did it work?”) and clarification (“why or how did it work?”) [70]. The literature is dominated by descriptive and justification studies that generally show SBE is effective [71]. This review shows that trend to be present in Sim-IPE in surgery. We did not find any clarification studies, which the SBE research community has been challenged to produce [1]. Such clarification studies require moving beyond research paradigms typical of the physical sciences and utilising educational theory and theoretical frameworks, all of which were rare in the studies included in this review [72]. There is a lack of studies that apply educational theories or frameworks to guide both design and evaluation. Without such theoretical grounding, it is unclear which constructs are being targeted, how change is expected to occur and whether results are generalisable across settings.

Outcomes were typically evaluated at Kirkpatrick level 1 (participant response) and 2 (acquisition of new knowledge, attitude or skills). This aligns with previous critiques of the SBE literature that too few studies evaluate changes in clinical practice and organisational or patient outcomes [71]. However, the studies [51, 52, 57, 73, 74] that did evaluate outcomes at Kirkpatrick level 4 show that with robust methodologies, longer follow up times and greater resourcing, such work is feasible. Similarly, a minority of studies showed that multi-site studies are also feasible despite the coordination challenges. Furthermore, multi-site Sim-IPE offers potential efficiency gains (e.g. reduced faculty time designing scenarios). Most importantly, multi-site implementation improves the robustness and generalisability of the findings. Weller (2020) exemplifies what is achievable with sustained high-level support and funding. New Zealand’s nation-wide trauma team Sim-IPE program (NetworkZ project), demonstrated improved system-level outcomes across multiple institutions [57].

The generally positive outcomes reported by interventional studies were unsurprising given the broader SBE literature and the likelihood of publication bias favouring positive findings. More interesting were the less intuitive findings. Apparent “negative” outcomes, such as reduced confidence after training, may represent productive recalibration of learners’ self-assessment rather than educational failure. Another notable finding was the benefit to observers. This aligns with social learning theory [75] and suggests simulation programs consider explicitly designing for observer engagement and not solely active participation [76]. Doing so provides the additional benefit of increased throughput of learners through the simulation program. Similarly, the use of Sim-IPE with students may offer an opportunity to shape collaborative attitudes early, before negative professional stereotypes become established. Finally, the incremental gains reported across repeated scenarios highlight an important unanswered question regarding the optimal “dose” of Sim-IPE [56].

Although rigorous evidence of its impact is limited, Sim-IPE is widely implemented in surgical training and many scenarios are highly complex. In situ simulation, despite being operationally demanding, is widely practiced [77, 78]. While in situ and ex situ simulation appear to offer similar learning benefits at the individual and team levels, in situ simulation may provide additional organisational learning, such as the identification of latent safety threats [77]. Additionally, while manikins have long been used in SBE [79], this review found that Sim-IPE in surgery scenarios often employed multiple simulation modalities allowing training in a broader range of skills and to a higher degree of quality. For example, Fiorentino (2021) conducted trauma resuscitation scenarios with manikin and augmented the simulation by having participants practise communication skills (breaking bad news to a family member) with simulated participants [80]. Finally, Sim-IPE’s flexibility is a major strength. Studies show Sim-IPE to provide educational solutions to novel challenges within surgical practice. Prominent examples include rapidly training staff in new protocols related to COVID-19 [51] and in facilitating the safe rollout of robotic surgery programs [81–83].

Studies described a rich variety of Sim-IPE scenarios – especially in emergency department and operating theatre settings and involving surgeons and critical care. Yet, gaps in practice were evident. Settings such as the intensive care unit and surgical ward were rarely represented and clinics, multidisciplinary meetings and the maternity ward not at all. Certain disciplines, notably radiology, were under-involved despite their importance in time-critical multidisciplinary decision-making. Obstetric contexts were sparsely represented within the reviewed literature, although this should be interpreted in the context of our exclusion of obstetrics and gynaecology as a primary specialty domain.

Our finding that skilled, multi-disciplinary faculty and institutional support are key facilitators for the implementation of effective and sustained Sim-IPE in surgery programs aligns with findings from the broader SBE literature [84, 85]. Sim-IPE is more than a “plug-and-play” educational novelty; it is resource-intensive and relies on organisational investment. This highlights the need for deliberate faculty development programs and strong institutional buy-in when commencing or scaling Sim-IPE in surgery. Barriers such as unprotected training time, unreliable funding, and cultural resistance to simulation as an educational modality are commonly reported in SBE [86, 87]. These issues may be amplified in surgery due to surgical service pressures (e.g. clinical utilisation of operating theatres) and traditional hierarchies (e.g. surgeon as “captain of the ship” rather than “member of the crew”) [88–90]. Overcoming these barriers will likely require both structural (policy, funding, scheduling) and cultural (leadership, team identity) change.

Many simulation scenarios were centred on the team performance of an action (e.g. trauma resuscitation in the emergency department or an operation in operating theatre). Typically, there was a clear expectation of how the action should be performed. Thus, the capacity for team decision-making was minimally challenged. However, in clinical practice, complex scenarios arise where there is uncertainty regarding the best course of action. Such scenarios increase the likelihood for interprofessional conflict, which is linked to poorer patient outcomes [91]. While such complex decision-making scenarios may occur in the emergency department, the operating theatre, intensive care unit, surgical ward or in multidisciplinary meetings, they could be simulated in easily accessible non-clinical spaces and with minimal equipment. We consider such simulations – focused on team communication and shared decision-making under uncertainty – to be underexplored in surgical Sim-IPE and a worthwhile area for future development.

While focused on surgery, the approach used in this review may be helpful to educators and researchers in other clinical domains as an example of how Sim-IPE can be characterised across settings, participants, modalities, implementation factors and evaluation methods.

Limitations of this review include restriction to English-language publications and the decision not to search grey literature. Given the heterogeneity of terminology in this field, some relevant studies describing teamwork or communication without explicit interprofessional descriptors may not have been captured by our search strategy. Further, we recognise practice may be advancing ahead of published research in this emerging field (the “theory-practice gap” [92]), so we have missed innovative Sim-IPE practices not yet reported in the literature. Finally, we note a transparency point: the published protocol focused on mapping Sim-IPE in surgery practice and research. During analysis a secondary objective to identify gaps and opportunities was added. This objective guided our thematic synthesis and recommendations reported here.

## Conclusion

This scoping review showed Sim-IPE in surgery is a new and rapidly expanding field. Studies described a rich variety of Sim-IPE scenarios, including multiple professions, disciplines, training levels, simulation modalities and settings, facilitating training in a broad range of skills reflective of the complexity of modern surgical care. The field needs more rigorous reporting, theory-driven design and implementation and higher-level evaluation to demonstrate organisational and patient benefits. Addressing the identified gaps – namely in multidisciplinary decision-making and under-represented settings – should be a priority for future practice and research.

## Data Availability

Data and materials pertaining to the study will be made available upon reasonable request to the corresponding author.

## Appendix 1

### Data charting

The data items charted were:

- Authors, year, country of publication, country of all authors, DOI Record type (e.g. interventional study, review, editorial, commentary, scenarios).
- Ethics approval (yes/no).
- Funding (academic/industry).
- Funding (academic/industry).
- Aims of the article/study.
- Study design.
- Inclusion criteria.
- Exclusion criteria.
- Description of intervention.
- Description of comparator group.
- Simulation modality (e.g. computer-based, manikin, simulated participants, role play etc.)
- Study participants:
  - ◦ Number.
  - ◦ Profession (e.g., doctor, nurse, allied health, other).
  - ◦ Discipline (e.g., emergency, intensive care, anaesthesia, surgery, medicine, radiology, other).
  - ◦ Training level (e.g., student, trainee, consultant, other).
- Setting (e.g. theatre, emergency department, ward, outpatient clinic, other).
- Educational theory.
- Theoretical frameworks.
- Outcome measures.
- Modified Kirkpatrick outcome evaluation level (44).
- Important results of interventional studies.
- Barriers and facilitators to implementation of Sim-IPE in surgery.

## Appendix 2

### Full references for included studies

1. Undre S, Koutantji M, Sevdalis N, Gautama S, Selvapatt N, Williams S, et al. Multidisciplinary crisis simulations: The way forward for training surgical teams. World Journal of Surgery. 2007;31 (9):1843-53.
2. Kozmenko V, Paige J, Chauvin S. Initial implementation of mixed reality simulation targeting teamwork and patient safety. Studies in health technology and informatics. 2008;132:216 − 21.
3. Paige JT, Chauvin SW. Transforming the Operating Room Team Through Simulation Training. Seminars in Colon and Rectal Surgery. 2008;19 (2):98–107.
4. Paige JT, Aaron DL, Yang T, Howell DS, Chauvin SW. Improved Operating Room Teamwork via SAFETY Prep: A Rural Community Hospital’s Experience. World Journal of Surgery. 2009;33 (6):1181-7.
5. Kinoshita T, Kanehira E, Matsuda M, Okazumi S, Katoh R. Effectiveness of a team participation training course for laparoscopy-assisted gastrectomy. Surgical Endoscopy and Other Interventional Techniques. 2010;24 (3):561-6.
6. Klipfel JM, Gettman MT, Johnson KM, Olson ME, Derscheid DJ, Maxson PM, et al. Using High-Fidelity Simulation to Develop Nurse-Physician Teams. Journal of Continuing Education in Nursing. 2011;42 (8):347 − 57.
7. Steinemann S, Berg B, Skinner A, Ditulio A, Anzelon K, Terada K, et al. In situ, multidisciplinary, simulation-based teamwork training improves early trauma care. Journal of Surgical Education. 2011;68 (6):472-7.
8. Ziewacz JE, Arriaga AF, Bader AM, Berry WR, Edmondson L, Wong JM, et al. Crisis checklists for the operating room: Development and pilot testing. Journal of the American College of Surgeons. 2011;213 (2):212-7.e10.
9. Stevens LM, Cooper JB, Raemer DB, Schneider RC, Frankel AS, Berry WR, et al. Educational program in crisis management for cardiac surgery teams including high realism simulation. Journal of Thoracic and Cardiovascular Surgery. 2012;144 (1):17–24.
10. Boet S, Bould MD, Sharma B, Revees S, Naik VN, Triby E, et al. Within-Team Debriefing Versus Instructor-Led Debriefing for Simulation-Based Education: A Randomized Controlled Trial. Annals of Surgery. 2013;258 (1):53 − 8.
11. Cumin D, Boyd MJ, Webster CS, Weller JM. A systematic review of simulation for multidisciplinary team training in operating rooms. Simulation in healthcare : journal of the Society for Simulation in Healthcare. 2013;8 (3):171-9.
12. Dedy NJ, Zevin B, Bonrath EM, Grantcharov TP. Current Concepts of Team Training in Surgical Residency: A Survey of North American Program Directors. Journal of Surgical Education. 2013;70 (5):578 − 84.
13. Seymour NE, Cooper JB, Farley DR, Feaster SJ, Ross BK, Pellegrini CA, et al. Best practices in interprofessional education and training in surgery: experiences from American College of Surgeons-Accredited Education Institutes. Surgery. 2013;154 (1):1–12.
14. Ziesmann MT, Widder S, Park J, Kortbeek JB, Brindley P, Hameed M, et al. S.T.A.R.T.T.: Development of a national, multidisciplinary trauma crisis resource management curriculum-Results from the pilot course. Journal of Trauma and Acute Care Surgery. 2013;75 (5):753-8.
15. Arora S, Cox C, Davies S, Kassab E, Mahoney P, Sharma E, et al. Towards the next frontier for simulation-based training: Full-hospital simulation across the entire patient pathway. Annals of Surgery. 2014;260 (2):252-8.
16. Arriaga AF, Gawande AA, Raemer DB, Jones DB, Smink DS, Weinstock P, et al. Pilot testing of a model for insurer-driven, large-scale multicenter simulation training for operating room teams. Annals of Surgery. 2014;259 (3):403 − 10.
17. Doumouras AG, Keshet I, Nathens AB, Ahmed N, Hicks CM. Trauma Non-Technical Training (TNT-2): the development, piloting and multilevel assessment of a simulation-based, interprofessional curriculum for team-based trauma resuscitation. Canadian journal of surgery. 2014;Journal canadien de chirurgie. 57 (5):354-5.
18. Huser AS, Muller D, Brunkhorst V, Kannisto P, Musch M, Kropfl D, et al. Simulated life-threatening emergency during robot-assisted surgery. Journal of Endourology. 2014;28 (6):717 − 22.
19. Paige JT, Garbee DD, Kozmenko V, Yu Q, Kozmenko L, Yang T, et al. Getting a head start: High-fidelity, simulation-based operating room team training of interprofessional students. Journal of the American College of Surgeons. 2014;218 (1):140-9.
20. Shapiro FE, Pawlowski JB, Rosenberg NM, Liu X, Feinstein DM, Urman RD. The use of in-situ simulation to improve safety in the plastic surgery office: a feasibility study. Eplasty. 2014;14:e2.
21. van der Nelson HA, Siassakos D, Bennett J, Godfrey M, Spray L, Draycott T, et al. Multiprofessional team simulation training, based on an obstetric model, can improve teamwork in other areas of health care. Am J Med Qual. 2014;29 (1):78–82.
22. Crozier MS, Ting HY, Boone DC, O’Regan NB, Bandrauk N, Furey A, et al. Use of human patient simulation and validation of the team situation awareness global assessment technique (TSAGAT): A multidisciplinary team assessment tool in trauma education. Journal of Surgical Education. 2015;72 (1):156 − 63.
23. Hamilton AJ, Prescher H, Biffar DE, Poston RS. Simulation trainer for practicing emergent open thoracotomy procedures. Journal of Surgical Research. 2015;197 (1):78–84.
24. Nicksa GA, Anderson C, Fidler R, Stewart L. Innovative approach using interprofessional simulation to educate surgical residents in technical and nontechnical skills in high-risk clinical scenarios. JAMA Surg. 2015;150 (3):201-7.
25. Paige JT, Garbee DD, Brown KM, Rojas JD. Using Simulation in Interprofessional Education. Surg Clin North Am. 2015;95 (4):751 − 66.
26. Phitayakorn R, Minehart RD, Hemingway MW, Pian-Smith MC, Petrusa E. The relationship between intraoperative teamwork and management skills in patient care. Surgery. 2015;158 (5):1434-40.
27. Stefanidis D, Ingram KM, Williams KH, Bencken CL, Swiderski D. Are Nursing Students Appropriate Partners for the Interdisciplinary Training of Surgery Residents? J Surg Educ. 2015;72 (5):823-8.
28. Weller J, Cumin D, Torrie J, Boyd M, Civil I, Madell D, et al. Multidisciplinary operating room simulation-based team training to reduce treatment errors: A feasibility study in New Zealand hospitals. New Zealand Medical Journal. 2015;128(1418):40–51.
29. Boet S, Pigford A-A, Fitzsimmons A, Reeves S, Triby E, Bould M. Interprofessional team debriefings with or without an instructor after a simulated crisis scenario: An exploratory case study. Journal of Interprofessional Care. 2016;30 (6):717 − 25.
30. Gillman LM, Widder S, Clément J, Engels PT, Paton-Gay JD, Brindley PG. Trauma simulation in bilingual Canada: Insurmountable barrier or unexpected strength? Insights from the first bilingual S.T.A.R.T.T. course. Can J Surg. 2016;59 (2):80 − 2.
31. Sadideen H, Weldon SM, Saadeddin M, Loon M, Kneebone R. A Video Analysis of Intra- and Interprofessional Leadership Behaviors Within “the Burns Suite”: Identifying Key Leadership Models. Journal of Surgical Education. 2016;73 (1):31 − 9.
32. Weller JM, Cumin D, Civil ID, Torrie J, Garden A, Maccormick AD, et al. Improved scores for observed teamwork in the clinical environment following a multidisciplinary operating room simulation intervention. New Zealand Medical Journal. 2016;129(1439):59–67.
33. Weller J, Civil I, Torrie J, Cumin D, Garden A, Corter A, et al. Can team training make surgery safer? Lessons for national implementation of a simulation-based programme. New Zealand Medical Journal. 2016;129(1443):9–17.
34. Davis WA, Jones S, Crowell-Kuhnberg AM, O’Keeffe D, Boyle KM, Klainer SB, et al. Operative team communication during simulated emergencies: Too busy to respond? Surgery. 2017;161 (5):1348-56.
35. Paige JT, Garbee DD, Yu Q, Rusnak V. Team Training of Inter-Professional Students (TTIPS) for improving teamwork. BMJ Simul Technol Enhanc Learn. 2017;3 (4):127 − 34.
36. Joshi K, Hernandez J, Martinez J, AbdelFattah K, Gardner AK. Should they stay or should they go now? Exploring the impact of team familiarity on interprofessional team training outcomes. Am J Surg. 2018;215 (2):243-9.
37. Lind MM, Corridore M, Sheehan C, Moore-Clingenpeel M, Maa T. A Multidisciplinary Approach to a Pediatric Difficult Airway Simulation Course. Otolaryngol Head Neck Surg. 2018;159 (1):127 − 35.
38. Sullivan S, Campbell K, Ross JC, Thompson R, Underwood A, LeGare A, et al. Identifying Nontechnical Skill Deficits in Trainees Through Interdisciplinary Trauma Simulation. Journal of Surgical Education. 2018;75 (4):978 − 83.
39. Wongsirimeteekul P, Mai CL, Petrusa E, Minehart R, Hemingway M, Pian-Smith M, et al. Identifying and Managing Intraoperative Arrhythmia: A Multidisciplinary Operating Room Team Simulation Case. MedEdPORTAL : the journal of teaching and learning resources. 2018;14:10688.
40. Jowsey T, Beaver P, Long J, Civil I, Garden AL, Henderson K, et al. Towards a safer culture: implementing multidisciplinary simulation-based team training in New Zealand operating theatres - a framework analysis. BMJ Open. 2019;9 (10):e027122.
41. Leithead J, 3rd, Garbee DD, Yu Q, Rusnak VV, Kiselov VJ, Zhu L, et al. Examining interprofessional learning perceptions among students in a simulation-based operating room team training experience. J Interprof Care. 2019;33 (1):26–31.
42. Long AM, Lefebvre CM, Masneri DA, Mowery NT, Chang MC, Johnson JE, et al. The Golden Opportunity: Multidisciplinary Simulation Training Improves Trauma Team Efficiency. J Surg Educ. 2019;76 (4):1116-21.
43. Paige JT. Developing Surgical Teams: Application. In: Nestel D, Dalrymple K, Paige JT, Aggarwal R, editors. Advancing Surgical Education: Theory, Evidence and Practice. Innovation and Change in Professional Education. 172019. p. 289–301.
44. Paige J, Garbee D, Yu Q, Kiselov V, Rusnak V, Detiege P. Moving Along: Team Training for Emergency Room Trauma Transfers (T (2) ERT (2)). J Surg Educ. 2019;76 (5):1402-12.
45. Rochlen LR, Malloy KM, Chang H, Kim S, Guichard L, Cassidy R, et al. Pilot One-Hour Multidisciplinary Team Training Simulation Intervention in the Operating Room Improves Team Nontechnical Skills. J Educ Perioper Med. 2019;21 (2):E624.
46. Weis JJ, Croft CL, Bhoja R, Lee JH, Scott DJ. Multidisciplinary Simulation Activity Effectively Prepares Residents for Participation in Patient Safety Activities. Journal of Surgical Education. 2019;76 (6):e232-e7.
47. Chheang V, Fischer V, Buggenhagen H, Huber T, Huettl F, Kneist W, et al. Toward interprofessional team training for surgeons and anesthesiologists using virtual reality. International Journal of Computer Assisted Radiology and Surgery. 2020;15 (12):2109-18.
48. Esparaz JR, Nierstedt RT, Elger BM, Chan VW, Evans CR, Egli SD, et al. Trauma Teams That Train as One Work as One: Invasive Procedure Training in Residency Education. Journal of Surgical Research. 2020;254:142-6.
49. Hoang TN, LaPorta AJ, Malone JD, Champagne R, Lavell K, De La Rosa GM, et al. Hyper-realistic and immersive surgical simulation training environment will improve team performance. Trauma Surg Acute Care Open. 2020;5 (1):e000393.
50. Long JA, Jowsey T, Henderson KM, Merry AF, Weller JM. Sustaining multidisciplinary team training in New Zealand hospitals: a qualitative study of a national simulationbased initiative. New Zealand Medical Journal. 2020;133(1516):10–21.
51. Lynch A. Simulation-based acquisition of non-technical skills to improve patient safety. Seminars in Pediatric Surgery. 2020;29 (2) (no pagination).
52. Mai CL, Wongsirimeteekul P, Petrusa E, Minehart R, Hemingway M, Pian-Smith M, et al. Prevention and Management of Operating Room Fire: An Interprofessional Operating Room Team Simulation Case. MedEdPORTAL : the journal of teaching and learning resources. 2020;16:10871.
53. Nataraja RM, Oo YM, Ljuhar D, Webb NR, Pacilli M, Win NN, et al. Overview of a novel paediatric surgical simulation-based medical education programme in Myanmar. ANZ journal of surgery. 2020;90 (10):1925-32.
54. Weller J, Long JA, Beaver P, Cumin D, Frampton C, Garden AL, et al. Evaluation of the effect of multidisciplinary simulation-based team training on patients, staff and organisations: protocol for a stepped-wedge cluster-mixed methods study of a national, insurer-funded initiative for surgical teams in New Zealand public hospitals. BMJ Open. 2020;10 (2):e032997.
55. Baste JM, Bottet B, Selim J, Sarsam M, Lefevre-Scelles A, Dusseaux MM, et al. Implementation of simulation-based crisis training in robotic thoracic surgery: how to improve safety and performance? Journal of Thoracic Disease. 2021;13.
56. Beaven A, Griffin D, James H. Highly realistic cadaveric trauma simulation of the multiply injured battlefield casualty: an international, multidisciplinary exercise in far-forward surgical management. Injury. 2021;52 (5):1183-9.
57. Caldwell KE, Lulla A, Murray CT, Handa RR, Romo EJ, Wagner JW, et al. Multi-Disciplinary Trauma Evaluation and Management Simulation (MD-TEAMS) training for emergency medicine and general surgery residents. American Journal of Surgery. 2021;221 (2):285 − 90.
58. Fiorentino M, Mosenthal AC, Bryczkowski S, Lamba S. Teaching Residents Communication Skills around Death and Dying in the Trauma Bay. Journal of Palliative Medicine. 2021;24 (1):77–82.
59. Gros E, Shi R, Hasty B, Anderson T, Schmiederer I, Roman-Micek T, et al. In situ interprofessional operating room simulations: Empowering learners in crisis resource management principles. Surgery. 2021;170 (2):432-9.
60. Huffman EM, Anton NE, Athanasiadis DI, Ahmed R, Cooper D, Stefanidis D, et al. Multidisciplinary simulation-based trauma team training with an emphasis on crisis resource management improves residents’ non-technical skills. Surgery. 2021;170 (4):1083-6.
61. Johnson GGRJ, Beaumont J, Paton-Gay JD, Widder S, Gillman LM. Multidisciplinary, multisite trauma team training during COVID-19: lessons from the first virtual E-S.T.A.R.T.T. course. Canadian journal of surgery. 2021;Journal canadien de chirurgie. 64 (6):E609-E12.
62. Kawaguchi AL, Kao LS. Teamwork and Surgical Team-Based Training. Surgical Clinics of North America. 2021;101 (1):15–27.
63. Lucke-Wold B, Gillham HE, Baskerville M, Cameron WE, Dillman D, Haley CA, et al. Establishing a Multidisciplinary Cavernous Carotid Injury Simulation to Train Neurosurgical, Otolaryngology, and Anesthesia Residents. J Vis Exp. 2021(175).
64. Lusty AJ, Bleackley J, Roberts M, Watterson J, Raiche I. Hemorrhaging laparoscopic partial nephrectomy - Feasibility of a novel simulation model. Canadian Urological Association Journal. 2021;16 (4) (no pagination).
65. Towning R, Rennie C, Ferguson M. Multidisciplinary Simulation Training for Surgical Tracheostomy in Patients Mechanically Ventilated Secondary to Severe Acute Respiratory Syndrome Coronavirus 2. Ear, Nose and Throat Journal. 2021.
66. Truong H, Sullivan AM, Abu-Nuwar MR, Therrien S, Jones SB, Pawlowski J, et al. Operating room team training using simulation: Hope or hype? American Journal of Surgery. 2021;222 (6):1146-53.
67. Dinh T, Khairallah T, Nguyen C, Deshpande G, Krzyzaniak S, Barton G, et al. PASS-A course in Pediatric Acute Surgical Support to build pediatric surgical emergencies capacity in developing countries. Journal of Pediatric Surgery. 2022;57 (12):1018-25.
68. Harrington J, Duncan G, K DA, Gable BD. Multidisciplinary Simulation Improves Resident Confidence for Pregnant Patients Requiring Surgical Intervention. Cureus. 2022;14 (3):e23454.
69. Hartman JA, Anderson DM, Ding J, Keech JC. Interprofessional veno-veno bypass simulation improved team confidence. Surgery. 2022;171 (4):904-7.
70. Hopmann P, Varre JS, Duncan G, Devoe WB, Gable BD. Multidisciplinary Simulation of Trauma in Pregnancy with Resuscitative Endovascular Balloon Occlusion of the Aorta (REBOA) Utilization. Cureus. 2022;14 (12):e32820.
71. Jogerst KM, Cassidy DJ, Coe TM, Monette D, Sell N, Eurboonyanum C, et al. Interprofessional Trauma Team Training: Leveraging Each Specialties’ Expertise to Teach Procedural-Based Skills. Journal of Surgical Education. 2022;79 (6):e273-e84.
72. Merritt-Genore H, Adams A, Zavala R, Brakke T. Interprofessional Simulation in Cardiothoracic Surgery Improves Team Confidence. Journal of Extra-Corporeal Technology. 2022;54 (3):250-4.
73. Picard J, Evain JN, Douron C, Maussion E, Stihle X, Manhes P, et al. Impact of a large interprofessional simulation-based training course on communication, teamwork, and safety culture in the operating theatre: A mixed-methods interventional study. Anaesthesia Critical Care and Pain Medicine. 2022;41 (1) (no pagination).
74. Sinyard RD, Rentas CM, Gunn EGM, Etheridge JC, Robertson JM, Gleason A, et al. Managing a team in the operating room: The science of teamwork and non-technical skills for surgeons. Current Problems in Surgery. 2022;59 (7).
75. Xue A, Chen S, Ranade A, Smith K, Kasten J, Catrip J, et al. How to implement a clinical robotic mitral valve surgery program. Annals of Cardiothoracic Surgery. 2022.
76. Escher C, Rystedt H, Creutzfeldt J, Meurling L, Hedman L, Felländer-Tsai L, et al. All professions can benefit - a mixed-methods study on simulation-based teamwork training for operating room teams. Adv Simul (Lond). 2023;8 (1):18.
77. Fitzgerald MC, Noonan M, Lim E, Mathew JK, Boo E, Stergiou HE, et al. Multi-disciplinary, simulation-based, standardised trauma team training within the Victorian State Trauma System. Emerg Med Australas. 2023;35 (1):62 − 8.
78. Gallagher J, Crockett M, McMeekin F. An emergency urology skills course for the multidisciplinary team. Journal of Clinical Urology. 2023;16 (3):252-8.
79. Horne EF, Thornton SW, Leraas HJ, O’Brian R, Greenwald E, Tracy ET. Use of pediatric trauma simulations to facilitate exposure to pediatric trauma resuscitations during training. Surgery. 2023.
80. Patki S, Nathan A, Lyness C, Nadarajah P, Sevastru S, Mahrous A, et al. Development of a high fidelity, multidisciplinary, crisis simulation model for robotic surgical teams. J Robot Surg. 2023;17 (5):2019-25.
81. Zucco L, Chen MJ, Levy N, Obeidat SS, Needham MJ, Hyatt A, et al. Just-in-Time In Situ Simulation Training as a Preparedness Measure for the Perioperative Care of COVID-19 Patients. Simulation in healthcare : journal of the Society for Simulation in Healthcare. 2023;18 (2):90 − 9.

## Appendix 3

### Modified Kirkpatrick level

**Table:**

Level 1: learners’ reaction
These outcomes relate to participants’ views of their learning experience and satisfaction with the program.
Level 2a: modification of attitudes/perceptions
Outcomes here relate to changes in reciprocal attitudes or perceptions between participant groups, towards patients/clients and their condition, circumstances, care and treatment.
Level 2b: acquisition of knowledge/skills
For knowledge, this relates to the acquisition of concepts, procedures and principles of interprofessional collaboration. For skills, this relates to the acquisition of thinking/problem-solving, psychomotor and social skills linked to collaboration.
Level 3: Change in behaviour
This level covers behavioural change transferred from the learning environment to the workplace prompted by modifications in attitudes or perceptions, or the application of newly acquired knowledge/skills in practice.
Level 4a: Change in organisational practice
This relates to wider changes in the organisation/delivery of care, attributable to an education program.
Level 4b: Benefits to patients/clients
This final level covers any improvements in the health and well-being of patients/clients as a direct result of an education program.

Reference:

Barr H, Freeth D, Hammick M, Koppel I, Reeves S. Evaluations of iterprofessional education. London: United Kingdom Review of Health and Social Care. 2000.

## Abbreviations

ACTIVE: Authors and Consumers Together Impacting on Evidence
COVID-19: Coronavirus disease of 2019
HPE: Health Professions Education
IPE: Interprofessional education
JBI: Joanna Briggs Institute
NOTSS: Non-technical Skills for Surgeons
PRISMA-ScR: Preferred Reporting Items for Systematic Reviews and Meta-Analyses Extension for Scoping Reviews
QI: Quality Improvement
SBE: Simulation-based education
Sim-IPE: Simulation-enhanced interprofessional education
USA: United States of America
